# Performance evaluation of *in vitro* diagnostic kits for hepatitis B virus infection using the regional reference panel of Japan

**DOI:** 10.1186/s12985-023-02054-7

**Published:** 2023-05-10

**Authors:** Haruka Momose, Asako Murayama, Norie Yamada, Keiji Matsubayashi, Sahoko Matsuoka, Emi Ikebe, Madoka Kuramitsu, Masamichi Muramatsu, Takanobu Kato, Isao Hamaguchi

**Affiliations:** 1 Research Center for Biological Products in the Next Generation, National Institute of Infectious Diseases, 4-7-1 Gakuen, Musashimurayama, Tokyo, 208-0011 Japan; 2 Department of Virology II, National Institute of Infectious Diseases, 1-23-1 Toyama, Shinjuku-ku, Tokyo, 162-8640 Japan; 3 Central Blood Institute, Blood Service Headquarters, Japanese Red Cross Society, 2-1-67 Tatsumi, Koto-ku, Tokyo, 135-8521 Japan

**Keywords:** Performance evaluation, *In vitro* diagnostics, HBV DNA, HBsAg, Reference panel

## Abstract

**Background:**

Hepatitis B virus (HBV) infection is a global public health concern. Precise and sensitive detection of viral markers, including HBV DNA and HBs antigen (Ag), is essential to determine HBV infection.

**Methods:**

The sensitivities and specificities of 5 HBV DNA and 14 HBsAg kits were evaluated using World Health Organization International Standards (WHO IS) and the Regional Reference Panel (RRP) consisting of 64 HBsAg-negative and 80 HBsAg-positive specimens.

**Results:**

All 5 HBV DNA kits detected HBV DNA in the WHO IS at a concentration of 10 IU/mL. The sensitivity and specificity to the RRP were 98.8–100% and 96.9–100%, respectively. HBV DNA titers were well correlated among the 5 kits regardless of HBV genotype. However, discordance of the HBV DNA titer was found in 5 specimens measured by CAP/CTM HBV v2.0. Among 12 automated HBsAg kits, the minimum detectable concentrations in the WHO IS varied from 0.01 to 0.1 IU/mL. Two lateral flow assays were positive for WHO IS concentrations greater than or equal to 1.0 and 0.1 IU/mL, respectively. When analyzed by the RRP, 12 automated kits exhibited a sensitivity of 98.8–100%, and 2 lateral flow assays showed sensitivities of 93.8% and 100%. The specificities of HBsAg kits were 100%. In the quantification of HBsAg, some kits showed a poor correlation of measurements with each other and showed up to a 1.7-fold difference in the regression coefficient of HBsAg titers. There were variations in the correlations of measurements among HBsAg kits when analyzed by genotype.

**Conclusions:**

Five HBV DNA kits showed sufficient sensitivity and specificity to determine HBV infection. HBV DNA titers were compatible with each other irrespective of HBV genotypes. HBsAg kits had enough sensitivity and specificity to screen for HBV infection. One of the lateral flow assays had a nearly equivalent sensitivity to that of the automated HBsAg kit. HBsAg titers quantified by the evaluated kits were not compatible across the kits. Genotype-dependent amino acid variations might affect the quantification of HBsAg titers.

**Supplementary Information:**

The online version contains supplementary material available at 10.1186/s12985-023-02054-7.

## Background

Hepatitis B is chronic hepatitis that is caused by infection with hepatitis B virus (HBV). Approximately 300 million people are infected by HBV worldwide [1] [2]. Without treatment, chronic HBV infection increases the risk of cirrhosis and liver cancer [3]. Therefore, precise and sensitive detection of HBV infection using reliable diagnosis kits is necessary.

Many diagnostic kits for HBV infections are available, including serological and molecular assays. Hepatitis B surface antigen (HBsAg) is a viral surface glycoprotein and is a useful serological marker for the screening and diagnosis of HBV infection. HBsAg is produced in infected hepatocytes and secreted into the blood. Thus, a high-titer of HBsAg indicates an active infection of HBV. Molecular tests for HBV DNA are used to confirm and define HBV infection. As molecular tests directly quantify the level of HBV DNA production, they are also used to monitor disease progression in chronic hepatitis B and the effectiveness of antiviral therapies [4].

A variety of diagnostics kits for HBV DNA and HBsAg are now available, so the correlation of the test results is important, including negative/positive decisions and measured values. The World Health Organization International Standards (WHO IS) are often utilized to standardize the measurements of each kit. However, the *in vitro* diagnostic kits may be affected by sequence variations of nucleotides or amino acids at the target regions [5] [6] [7] [8]. The diversity of sequences is characterized by predominant genotypes or strains in each geographic region. Since the standardization by use of WHO IS guarantees the results of only one genotype, the evaluation of diagnostics kits using specimens of endemic strains is essential. For this purpose, we have established the regional reference panel (RRP) consisting of 64 HBsAg-negative and 80 HBsAg-positive specimens derived from blood donors in Japan. The RRP has been updated regularly because predominant genotypes or strains may change with time. In Japan, the infections of genotypes (GT) B and C are prevalent in chronic hepatitis B patients who are infected at birth or during infancy [9] [10], and the infection of GTA has increased in acute hepatitis B in adults [11] [12]. Diagnostics kits for HBV infection are expected to detect or quantify the titer of HBV strains of such genotypes equally well. The RRP used in this study includes HBV GTA, GTB, GTC, and GTD which are predominant in Japan. This RRP is also used to assess the correlations of titers of HBV DNA and HBsAg quantification kits. In Japan, when releasing a new kit, the regulatory authorities request that it perform as well as or better than existing kits on the market. The regression coefficient (slope of the correlation equation) of quantified values between the new and existing kits should be between 0.9 and 1.1, and the coefficient of determination (R^2^) for these kits should exceed 0.9. In this study, by using this RRP and the WHO IS for HBV DNA and HBsAg, 5 HBV DNA quantification kits and 14 HBsAg detection/quantification kits were evaluated.

## Methods

### The WHO ISs for HBV DNA and HBsAg

The 4th WHO IS for HBV DNA (NIBSC code: 10/266) was obtained from the National Institute of Biological Standards and Control (NIBSC, UK). It comprises plasma specimen from an HBV carrier who was persistently HBsAg and hepatitis B e antigen-positive (GTA2) [13] [14]. The assigned titer of this WHO IS is 955,000 International Units (IU)/mL. The 3rd WHO IS for HBsAg (NIBSC code: 12/226) was also obtained from the NIBSC. It contains plasma from asymptomatic carriers positive for HBsAg, which was purified and inactivated for the manufacture of a plasma-derived hepatitis B vaccine (GTB4) [15]. The assigned titer of this WHO IS is 47.3 IU/mL. These WHO ISs were serially diluted and stored at -80 °C until use.

### Establishment of the RRP

For the establishment of the RRP, 64 HBsAg-negative and 80 HBsAg-positive plasma specimens were provided by the Japanese Red Cross Blood Center. These specimens were from blood donors and were collected from 2013 to 2015 in Japan. Lumipulse Presto HBsAg-N (FUJIREBIO INC., Tokyo, Japan) was used to determine HBsAg for these specimens. The specimens were aliquoted in 1.0 mL volumes into 1.5 mL screw-cap tubes after centrifugation at 3,000 rpm for 10 min to exclude agglutinates or clots and stored at -80 °C until further use.

### Genotyping of HBV in RRP

**Fig. 1:**
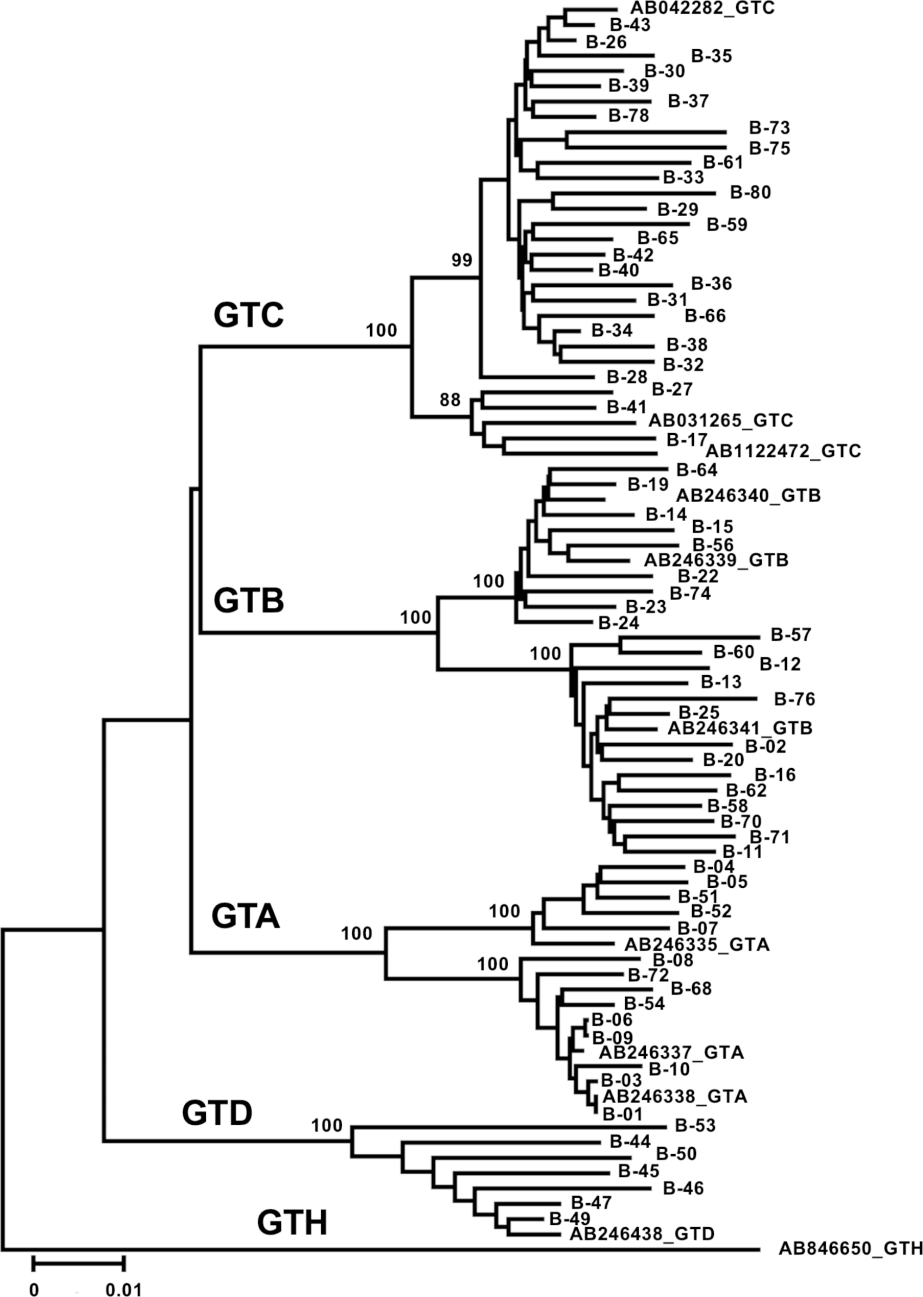
Phylogenetic analysis of HBV isolated in this study The whole genome sequences of HBV in RRP HBsAg-positive specimens were determined, and the genotypes of HBV were determined by phylogenetic analysis. The representative strains in each genotype are included in this tree with an accession number

Viral DNA was extracted with the QIAsymphony DSP Virus/Pathogen midi kit (QIAGEN, Valencia, CA) from 1 mL of plasma. The whole genome of HBV in each specimen was amplified in two fragments A and B with the following primers (for the 1st round PCR of fragment A; forward, 5’-ATTCCACCAAGCTCTGCTAGATCCCAGAGT-3’; reverse, 5’-GGTGCTGGTGAACAGACCAATTTATGCCTA-3’; for the 2nd round PCR of fragment A; forward, 5’-CCTATATCTTCCTGCTGGTGGCTCCAGTTC-3’; reverse, 5’-TAACCTAATCTCCTCCCCCAACTCCTCCCA-3’; for the 1st round PCR of fragment B; forward, 5’-ACGTCGCATGGAGACCACCGTGAACGCCCA-3’; reverse, 5’-AAGTCCACCACGAGTCTAGACTCTGTGGTA-3’; for the 2nd round PCR of fragment B; forward, 5’-CATGGTCTTGCCCAAGGTCTTGCATAAGAG-3’; reverse, 5’-CCCGCCTGTAACACGAGCAGGGGTCCTAGG-3’). The amplified products were sequenced using primers reported by Chook et al. [16]. HBV genotypes were determined by phylogenetic analysis with a representative strain of each genotype (Fig. 1). Specimens that could not be amplified were genotyped by IMMUNIS HBV Genotype EIA (Institute of Immunology Co., Ltd., Tokyo, Japan) [17].

### Evaluation of ***in vitro*** diagnostic kits for HBV DNA and HBsAg

Five HBV DNA quantification kits and 14 HBsAg quantification/detection kits were evaluated in this study. The 5 HBV DNA quantification kits were Abbott Real-Time HBV test (ART HBV; Abbott Japan, Tokyo, Japan), Alinity m HBV Assay (Alinity HBV; Abbott Japan), Cobas AmpliPrep/Cobas TaqMan HBV test version 2.0 (CAP/CTM HBV v2.0; Roche Diagnostics, Tokyo, Japan), Cobas 6800/8800 system HBV (Cobas HBV; Roche Diagnostics), and Aptima HBV Quant Assay (Aptima HBV; Hologic, Inc., Tokyo, Japan). For HBsAg kits, 8 quantification kits, Lumipulse HBsAg-HQ (Lumipulse; FUJIREBIO INC., Tokyo, Japan), Lumipulse Presto HBsAg-HQ (Presto; FUJIREBIO INC.), HISCL HBsAg Assay Kit (HISCL; Sysmex Co., Kobe, Japan), CL AIA-PACK HBsAg (CL AIA; Tosoh Co., Tokyo, Japan), Accuraseed HBsAg (Accuraseed; FUJIFILM Wako Pure Chemical Corporation, Osaka, Japan), Architect HBsAg QT (Architect; Abbott Japan), Alinity HBsAg QT (Alinity; Abbott Japan) and Elecsys HBsAg II quant II (Elecsys quant II; Roche Diagnostics), and 4 detection kits, ST AIA-PACK HBsAg (ST AIA; Tosoh Co.), STACIA CLEIA HBsAg (STACIA; LSI Medience Corp., Tokyo, Japan), ADVIA Centaur HBsAg II (Centaur II; Siemens Healthcare Diagnostics K.K., Tokyo, Japan) and Elecsys HBsAg II (Elecsys II; Roche Diagnostics), were enrolled. All assays were performed by the respective manufacturers at their research laboratories. In addition to 12 automated HBsAg assays, 2 lateral flow assays (LFAs) for HBsAg, Determine HBsAg (Determine; Abbott Diagnostics Medical Co., Tokyo, Japan) and Determine HBsAg 2 (Determine2; Abbott Diagnostics Medical Co.), were also enrolled. These kits were evaluated in our laboratory using kits provided by the manufacturer.

### Statistical analysis

Statistical analysis was performed in GraphPad Prism 9 (GraphPad Software, La Jolla, CA) to determine the correlations of quantitative data using Pearson’s correction coefficient analysis, and the R^2^ value was calculated.

## Results

### Genotype distribution of HBsAg-positive specimens in RRP

The RRP includes 64 HBsAg-negative (BN-1 – BN-64) and 80 HBsAg-positive (B-1 – B-80) plasma specimens. To determine HBV genotypes, DNA was extracted from HBsAg-positive specimens, and the whole HBV genome was amplified by PCR and sequenced. The HBV genomes of 71 isolates were sequenced and genotyped by phylogenetic analysis. Fourteen specimens were identified as GTA, 23 as GTB, 27 as GTC, and 7 as GTD (Fig. 1). HBV genotypes of the remaining 8 specimens that were not amplified by PCR were determined by IMMUNIS HBV Genotype EIA: 1 as GTA, 4 as GTB, 2 as GTC, and 1 as GTD (data not shown). One specimen, B-69, could not be genotyped by any methods. Overall, the positive panel included HBV GTA (15; 18.8%), GTB (27; 33.8%), GTC (29; 36.3%), GTD (8; 10.0%), and undetermined (1; 1.3%).

### Evaluation of HBV DNA kits by WHO IS

The performances of 5 HBV DNA kits were evaluated with the WHO IS for HBV DNA (DNA-IS) (Table 1). ART HBV and CAP/CTM HBV v2.0 have been used in clinical practice in Japan for a decade. Alinity HBV, Cobas HBV, and Aptima HBV were recently introduced into the market. Target regions of ART HBV, Alinity HBV, and Aptima HBV are in the HBs region, whereas target regions of CAP/CTM HBV v2.0 and Cobas HBV are in the precore/core (preC/C) region (Additional file 1: Table S1). These kits’ quantification results of serially diluted DNA-IS were close to the theoretical titers (Table 1). The values of the coefficient of variation (CV) were below 10% in the quantification of high-titer specimens DNA-IS 1 to DNA-IS 4 with amounts over 2.5 log IU/mL. The CV values were often beyond 10% in the quantification of the low-titer specimens by several kits, DNA-IS 5 to DNA-IS 7, with amounts under 2.0 log IU/mL. However, all kits determined DNA-IS 7 with a concentration of 10 IU/mL as positive. The Diluent of DNA-IS was determined to be negative by all kits (data not shown).

**Table 1:** Titers of the WHO IS for HBV DNA quantified by HBV DNA kits

DNA-IS		DNA-IS 1	DNA-IS 2	DNA- IS 3	DNA- IS 4	DNA- IS 5	DNA- IS 6	DNA- IS 7
Theoretical titer (log IU/mL)		4.0	3.5	3.0	2.5	2.0	1.5	1.0
ART HBV	Titer (log IU/mL)	3.85	3.39	2.94	2.45	2.05	1.77	1.19
	CV (%)	3.75	3.14	2.00	2.00	2.50	18.0	19.0
Alinity HBV	Titer (log IU/mL)	3.99	3.52	3.11	2.62	2.26	1.56	1.34
	CV (%)	0.25	0.57	3.67	4.80	13.0	4.00	34.0
CAP/CTM HBV v2.0	Titer (log IU/mL)	4.03	3.53	3.00	2.43	2.11	1.46	< 1.30
	CV (%)	0.75	0.86	0.00	2.80	5.50	2.67	ND
Cobas HBV	Titer (log IU/mL)	3.92	3.41	2.93	2.47	1.99	1.49	1.09
	CV (%)	2.00	2.57	2.33	1.20	0.50	0.67	9.00
Aptima HBV	Titer (log IU/mL)	4.04	3.57	3.11	2.56	2.30	1.51	1.20
	CV (%)	1.00	2.00	3.67	2.40	15.0	0.67	20.0

### Evaluation of HBV DNA kits by RRP

These kits were also evaluated by detection and quantification of HBsAg-negative (n = 64) and HBsAg-positive (n = 80) specimens in the RRP. In the evaluation of HBsAg-negative specimens, all specimens were determined to be negative by ART HBV, Alinity HBV, and Aptima HBV. On the other hand, CAP/CTM HBV v2.0 and Cobas HBV determined 2 specimens (BN-9 and BN-14) and 1 specimen (BN-14) as positive, respectively. The specificities of these kits were calculated to be 96.9% and 98.4%, respectively (Table 2).

**Table 2:** Results of HBV RRP specimens detected by HBV DNA kits

HBV DNA kits	HBV RRP		
	Negative (n = 64)	Positive (n = 80)	
	Tested Negative	Tested Positive	Within quantitative range
ART HBV	64 (100%)	80 (100%)	77 (96.3%)
Alinity HBV	64 (100%)	79 (98.8%)	77 (96.3%)
CAP/CTM HBV v2.0	62 (96.9%)	79 (98.8%)	72 (90.0%)
Cobas HBV	63 (98.4%)	79 (98.8%)	76 (95.0%)
Aptima HBV	64 (100%)	80 (100%)	72 (90.0%)

**Fig. 2:**
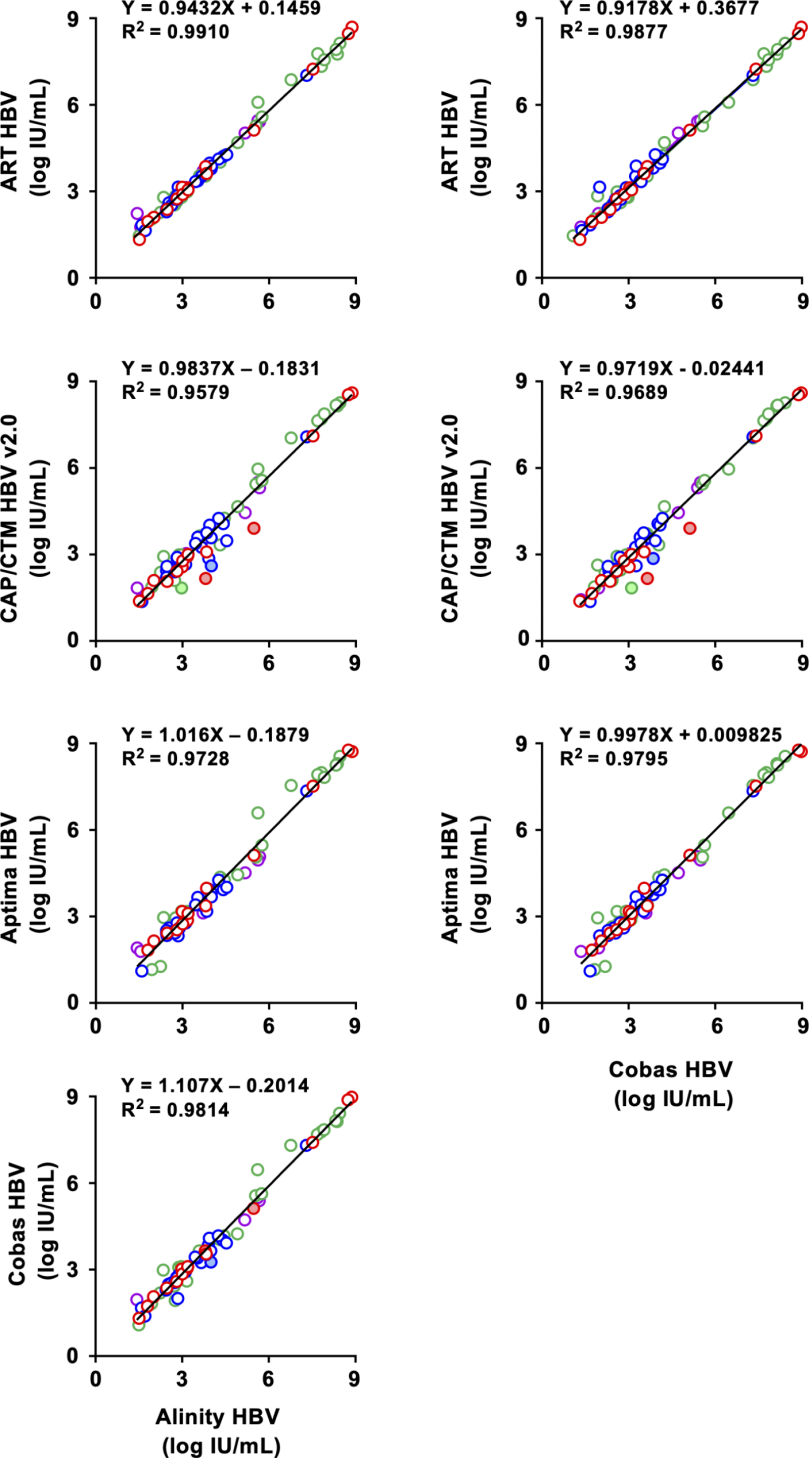
Correlation of HBV DNA titers HBsAg-positive specimens of RRP were quantified by HBV DNA kits. HBV DNA titers were compared with Alinity HBV and Cobas HBV. Data and linear regression lines are shown in dots and lines, respectively. Red; GTA, blue; GTB, green; GTC, purple; GTD.

In the evaluation of HBsAg-positive specimens, the detection rate of these kits ranged from 98.8 to 100%. ART HBV and Aptima HBV detected HBV DNA in all specimens. CAP/CTM HBV v2.0, Alinity HBV, and Cobas HBV failed to detect the HBV DNA in 1 specimen that indicated low titer, although the specimen determined as negative was different (B-18 by CAP/CTM HBV v2.0, and B-77 by Alinity HBV and Cobas HBV). In the quantification by these kits, the number of quantified specimens was different among the kits (Table 2). ART HBV and Alinity HBV quantified 77 specimens (96.3%), followed by Cobas HBV (76 specimens; 95.0%), CAP/CTM HBV v2.0 (72 specimens; 90.0%), and Aptima HBV (72 specimens; 90.0%). The HBV DNA titers quantified by these kits showed good correlations with each other (Fig. 2). For example, in the correlation between the titers by Alinity HBV and by ART HBV, CAP/CTMHBV v2.0, and Aptima HBV, the slopes of linear regressions ranged from 0.9432 to 1.016. Likewise, similar data were observed in the correlation between the titers by Cobas HBV and by ART HBV, CAP/CTMHBV v2.0, and Aptima HBV. The correlation between the titers by Alinity HBV and Cobas HBV was also good, and the slope of linear regression was 1.107 (Fig. 2). Genotype-dependent differences were not detected in these correlations. Although the R^2^ values were over 0.95 for all kits, the R^2^ values of CAP/CTM HBV v2.0 were low compared to those of other kits, which were 0.9579 and 0.9689 in correlations with Alinity HBV and Cobas HBV, respectively. To clarify the reason for these low R^2^ values in the correlation between CAP/CTM HBV v2.0 and the other kits, the quantified titers were analyzed in Bland‒Altman plots. In the correlation between Alinity and CAP/CTM HBV v2.0, 4 specimens (B-7, B-33, B-57, and B-68) were shown to deviate. In the correlation between Cobas HBV and CAP/CTM HBV v2.0, 4 specimens (B-7, B-11, B-33, and B-68) were shown to deviate from the correlation (Additional file 1: Figs. S1A and S1B). The deviations of these 5 specimens (B-7, B-11, B-33, B-57, and B-68) in HBV DNA titers quantified by CAP/CTM HBV v2.0 were not observed in the quantification by Cobas HBV, which was newly released by the same manufacturer, and the R^2^ value was 0.9814 between Alinity HBV and Cobas HBV (Fig. 2).

### Evaluation of HBsAg kits by WHO IS and RRP

Fourteen HBsAg kits were evaluated as listed in Additional file 1: Table S2. Among the 12 automated HBsAg kits, 8 kits were quantitative, and 4 kits were qualitative. The WHO IS for HBsAg (HBs-IS) was serially diluted as indicated and subjected to detection and quantification by these HBsAg kits (Table 3). Among 12 kits that use a fully automated system, the maximum dilution factor of HBs-IS that could be detected was different among the kits. Lumipulse and Presto exhibited the highest sensitivity and quantified the HBs-IS with a theoretical concentration of 0.01 IU/mL (HBs-IS 6) as the lowest concentration. Other automated kits, regardless of whether they were quantitative or qualitative, detected the HBs-IS with a theoretical concentration of 0.1 (HBs-IS 4) or 0.03 (HBs-IS 5) IU/mL as the lowest concentration.

**Table 3:** Evaluation of HBsAg kits by WHO IS.

HBs-IS		HBs-IS 1		HBs-IS 2		HBs-IS 3		HBs-IS 4		HBs-IS 5	HBs-IS 6	HBs-IS 7
Theoretical titer (IU/mL)		3.0		1.0		0.3		0.1		0.03	0.01	0.003
Quantitative	Lumipulse (IU/mL)	3.021		1.017		0.309		0.099		0.032	0.010	[0.003]
	Presto (IU/mL)	3.206		1.111		0.330		0.111		0.034	0.011	[0.003]
	HISCL (IU/mL)	3.41		1.12		0.34		0.10		0.03	[0.01]	[0.00]
	CL AIA (IU/mL)	2.54		0.90		0.27		0.10		0.03	[0.01]	[0.01]
	Accuraseed (IU/mL)	3.66		1.16		0.35		0.12		0.05	[0.00]	[0.00]
	Architect (IU/mL)	4.20		1.46		0.43		0.14		[0.03]	[0.00]	[0.00]
	Alinity (IU/mL)	4.15		1.40		0.49		0.16		0.05	[0.02]	[0.00]
	Elecsys quant II (IU/mL)	2.93		0.944		0.279		0.0859		[0.05 <]	[0.05 <]	[0.05 <]
Qualitative	ST AIA (IU/mL)	2.66		0.86		0.25		0.08		[0.03]	[0.01]	[0.00]
	STACIA (COI)	60.9		20.7		6.1		2.0		[0.6]	[0.2]	[0.1]
	Centaur II (Index)	204.05		63.42		17.03		5.66		1.61	[0.55]	[0.26]
	Elecsys II (COI)	56.6		19.7		6.08		2.27		1.05	[0.648]	[0.53]
LFA	Determine (+/-)	+		+		-		-		-	-	-
	Determine2 (+/-)	+		+		+		+		-	-	-

The titer in brackets indicates the value under the detection limit

The specificities of HBsAg assays were evaluated with RRP (Table 4). All negative specimens were determined to be seronegative by these kits, and no positives were observed. Thus, the specificity of these HBsAg kits in the evaluation with RRP is 100%. In the evaluation of positive specimens in RRP, the sensitivities of most automated HBsAg kits were 100%. A few qualitative kits failed to detect HBsAg in 1 specimen, and the sensitivities were 98.8%.

**Table 4:** HBV RRP evaluated by HBsAg kits

HBsAg kits		HBV RRP	
		Negative (n = 64) Tested Negative	Positive (n = 80) Tested positive
Quantitative	Lumipulse	64 (100%)	80 (100%)
	Presto	64 (100%)	80 (100%)
	HISCL	64 (100%)	80 (100%)
	CL AIA	64 (100%)	80 (100%)
	Accuraseed	64 (100%)	80 (100%)
	Architect	64 (100%)	80 (100%)
	Alinity	64 (100%)	80 (100%)
	Elecsys quant II	64 (100%)	80 (100%)
Qualitative	ST AIA	64 (100%)	79 (98.8%)
	STACIA	64 (100%)	79 (98.8%)
	Centaur II	64 (100%)	80 (100%)
	Elecsys II	64 (100%)	80 (100%)
LFA	Determine	64 (100%)	75 (93.8%)
	Determine2	64 (100%)	80 (100%)

Two LFA kits, Determine and Determine2, were also evaluated by serially diluted HBs-IS and RRP (Tables 3 and 4). Determine detected HBs-IS with theoretical concentrations of 3 and 1 IU/mL. Of note, Determine2 could detect the HBs-IS with a theoretical concentration of 0.1 IU/mL as the lowest concentration. The detection rates of Determine and Determine2 were 93.8% and 100%, respectively, when examined in the RRP.

### Quantification of HBsAg titers

**Fig. 3:**
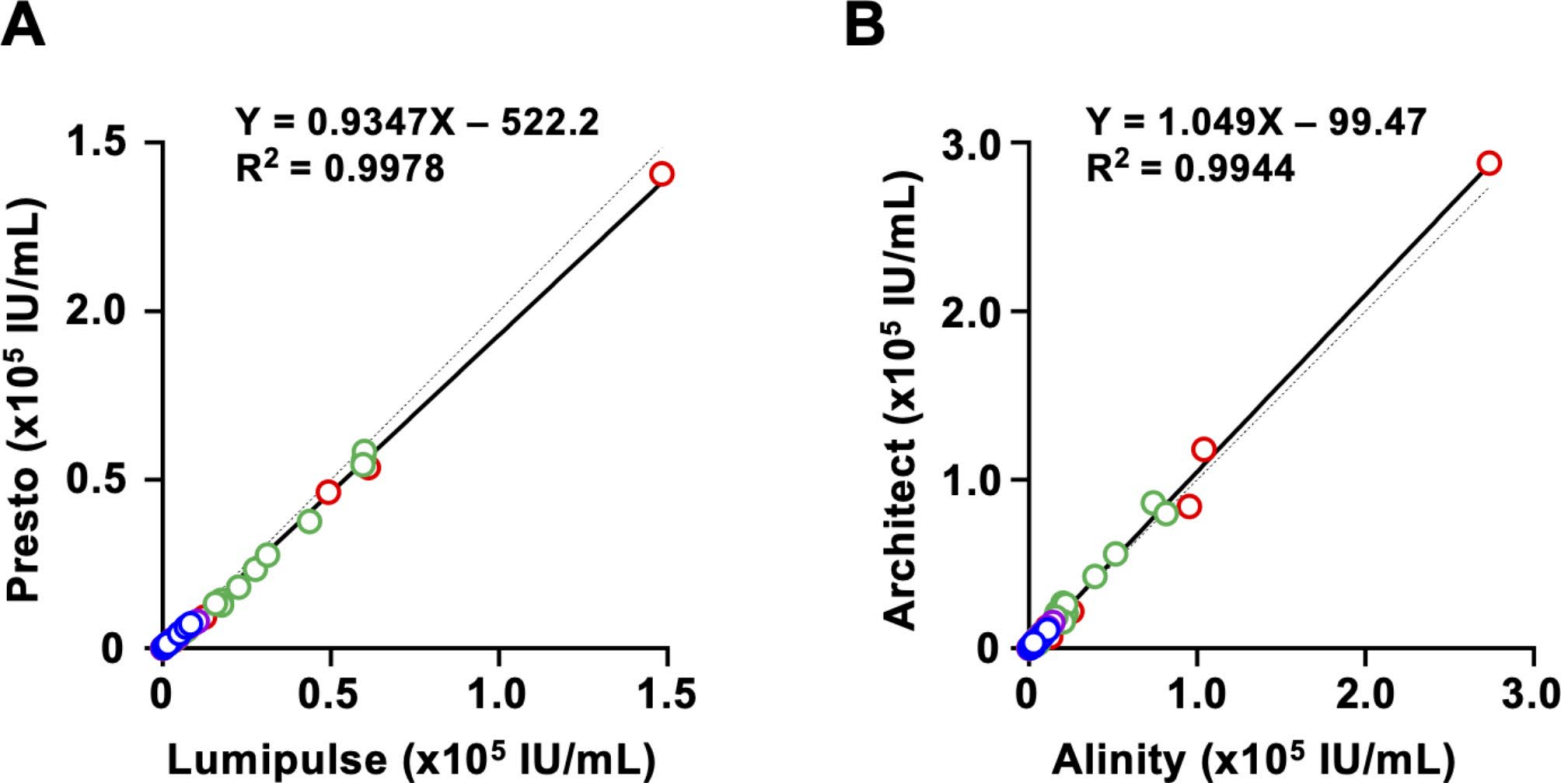
Correlations of HBsAg titers between kits from the same manufacturers Correlations of HBsAg titers were analyzed between FUJIREBIO **(A)** and Abbott **(B)** kits. Data and linear regression lines are shown in dots and lines, respectively. Red; GTA, blue; GTB, green; GTC, purple; GTD. Dashed lines indicate a slope of 1.0

The quantified HBsAg titers of specimens in the RRP were compared among 8 HBsAg quantification kits. High titer specimens over the dynamic range of each kit were diluted and quantified. First, the HBsAg titers quantified by HBsAg kits released by the same manufacturer were compared. Between FUJIREBIO kits (Lumipulse vs. Presto), quantified titers showed a good correlation: the slope of linear regression was 0.9347, and the R^2^ value was 0.9978 (Fig. 3A). A similar good correlation was observed between Abbott kits (Alinity vs. Architect); the slope of linear regression was 1.049, and the R^2^ value was 0.9944 (Fig. 3B).

**Fig. 4:**
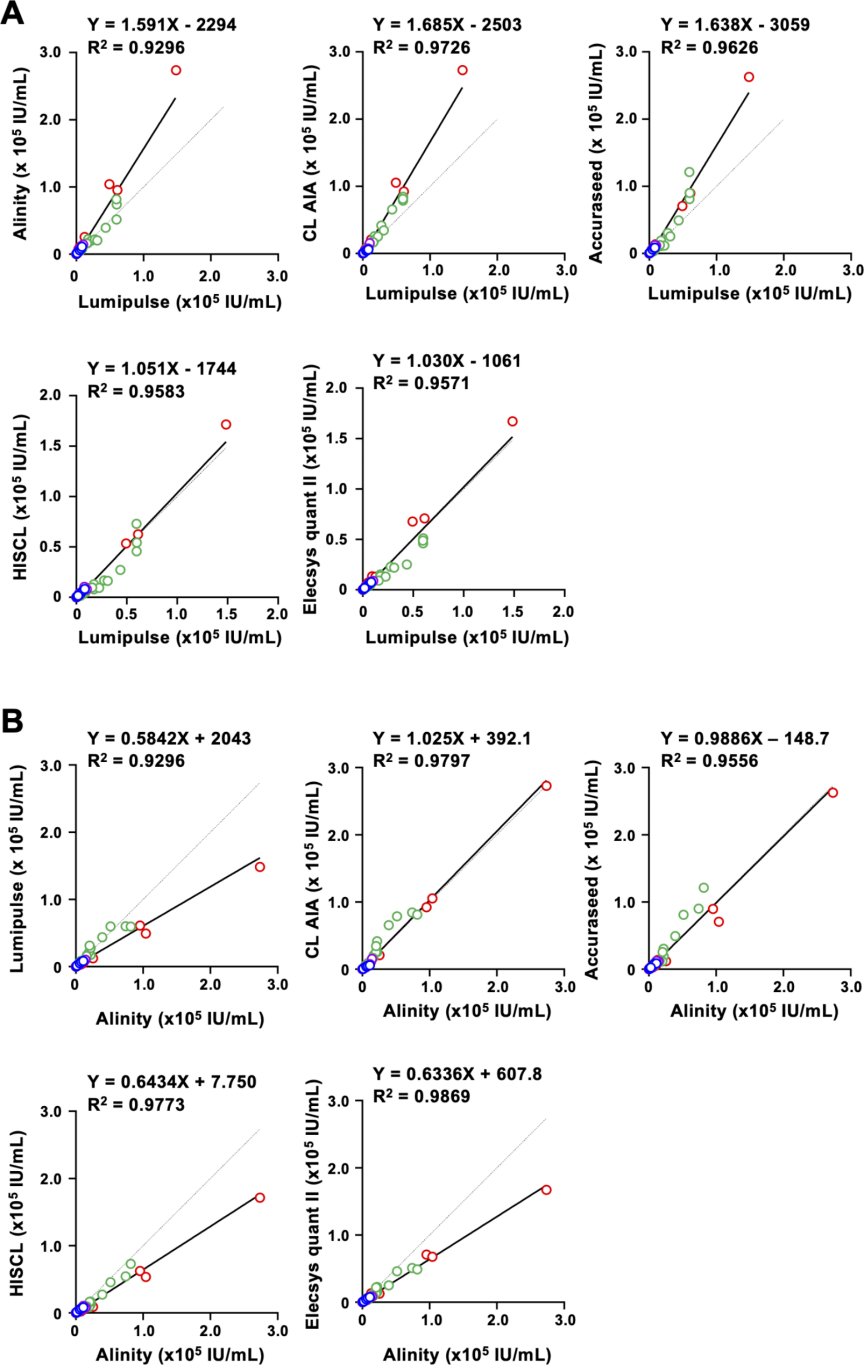
Correlations of HBsAg titers between kits of different manufacturers Correlations of HBsAg titers of HBsAg quantitative assays were compared with Lumipulse **(A)** and Alinity **(B)**. Data and linear regression lines are shown in dots and lines, respectively. Red; GTA, blue; GTB, green; GTC, purple; GTD. Dashed lines indicate a slope of 1.0

On the other hand, between kits released by different manufacturers, no good correlation of quantified titers was obtained. When comparing the HBsAg titers quantified by Lumipulse with Alinity, the slope of the linear regression was 1.591, which was far from 1.0 (Fig. 4A). Linear regression slopes over 1.5 were also observed in the correlations to CL AIA and Accuraseed. In addition, the slopes of the linear regression varied among genotypes (Additional file 1: Fig. S2). In the correlation between Lumipulse and Alinity, the highest value of the slope was observed in titers of GTA (1.832), and the lowest value was observed in titers of GTC (1.082). Such genotype dependency was also observed in the correlation with other kits.

When comparing the HBsAg titers quantified by Alinity with other kits, the slope of the linear regression was low (Fig. 4B). The linear regression slopes for 3 of the 5 kits, Lumipulse, HISCL, and Elecsys quant II, were below 0.65. Genotype dependencies were also detected in correlations between Alinity and other kits (Additional file 1: Fig. S3). The slopes to the 3 kits, Lumipulse, HISCL, and Elecsys quant II, were varied but less than 1.0 for all genotypes. In the correlation to CL AIA, the slope indicates a good correlation and was 1.025 when assessed with all specimens. When assessed with each genotype, the slopes of GTA and GTD were within 0.9 to 1.1, but the slopes of GTB and GTC were 0.5776 and 1.201, respectively. Similarly, in the correlation to Accuraseed, a good correlation was shown with a slope of 0.9886 when assessed with all specimens, and the slopes of GTA, GTB, and GTD indicated similar values, whereas the slope of GTC was 1.413, which was far outside the range of 0.9–1.1. Similar genotype dependencies in correlations of HBsAg titers were also confirmed when comparing the HBsAg titers quantified by Elecsys quant II with other kits (Additional file 1: Fig. S4).

## Discussion

Accurate and reliable diagnostic kits for HBV DNA and HBsAg are crucial for diagnosing HBV infection. For the evaluation of these kits, appropriate calibrators such as WHO IS and a panel of regional specimens are necessary. In this study, we established the RRP for HBV consisting of plasma specimens from blood donors in Japan. This RRP contains all predominant HBV genotypes that emerged in Japan [9] [10] and is useful to evaluate whether the kits for HBV DNA and HBsAg can correctly analyze the specimens of Japanese patients [8].

The precise detection of HBV DNA is important for the diagnosis of HBV infection. Of the 5 HBV DNA kits evaluated in this study, 2 kits, CAP/CTM HBV v2.0 and Cobas HBV, detected HBV DNA from the 2 HBsAg-negative specimens in the RRP. These kits were released by the same manufacturer and target the preC/C region, which is different from other HBV DNA kits. This observation may indicate that a small amount of HBV DNA fragments of the preC/C region but not the full genome of HBV is included in these specimens. We considered these specimens as inappropriate for the evaluation of HBV DNA kits and decided to exclude them from the RRP. Regarding sensitivity, all kits tested positive for DNA-IS 7 (10 IU/mL) and were considered as sufficiently sensitive. Consistently, the positive rates of the RRP by these HBV DNA kits ranged from 98.8 to 100%. Three kits failed to detect HBV DNA in 1 HBsAg-positive specimen in the RRP. The specimens that tested negative were varied by kits, and the titers in these specimens were less than 10 IU/mL. As seen from the DNA-IS data, low-titer specimens tended to have larger CV values and lower accuracy. These data suggest that HBV DNA kits may fail to detect HBV DNA in specimens at concentrations around the lower detection limits. The titer of HBV DNA is also important to understand the prognosis and to evaluate the therapeutic effects of chronic hepatitis B [3]. The quantified HBV DNA titers of HBsAg-positive specimens in the RRP showed good correlations among these kits regardless of HBV genotypes [18] [19]. Exceptionally, deviation of HBV DNA titers of 5 specimens was shown in CAP/CTM HBV v2.0. It is suggested that there may be mutations in the target region of CAP/CTM HBV v2.0 in these 5 specimens [20]. These deviations were resolved in Roche’s new kit Cobas HBV. Taken together, the evaluated HBV DNA kits, especially those released recently, are considered to have sufficient specificities and sensitivities for the diagnosis of HBV infection in clinical practice. The measured HBV titers were compatible with each other and were not affected by the HBV genotypes.

HBsAg is an effective marker for screening for HBV infection. We found that the sensitivities of 12 automated HBsAg kits evaluated in this study were different by more than 10-fold in the evaluation by HBs-IS. In the evaluation with the RRP, the detection rates of these kits were shown to be 98.8–100%. Therefore, despite differences in detection sensitivity, 12 HBsAg kits were found to be usable for screening for HBV infection. In addition to 12 automated assays, 2 LFA kits, Determine and Determine2, were evaluated in this study. LFA kits are known to need no power supply and are easy to use with low cost but are considered to have the disadvantage of low sensitivity [21]. However, in this study, Determine2 exhibited high sensitivity. This kit detected HBs-IS at a concentration of 0.1 IU/mL and tested positive for all positive RRP specimens. These results indicated that Determine2 has equivalent sensitivity to that of automated HBsAg kits and can be used in screening for HBV infections in low-resource settings [22].

HBsAg levels in the blood specimens of hepatitis B patients are used to understand disease progression and monitor the effectiveness of antiviral therapies [23] [24]. Thus, the HBsAg titers quantified by the various kits are expected to be the same. In this study, good correlations of HBsAg titers were found for kits from the same manufacturer. Manufacturers producing more than 1 HBsAg kit use the same or similar antibodies for their own kits, which leads to a good correlation in the measured values of HBsAg. On the other hand, no good correlations were found for HBsAg kits that were released by different manufacturers. The linear regression analysis suggested that the HBsAg titers quantified by Lumipulse are often low compared to the titers quantified by Alinity, CL AIA, and Accuraseed. Similarly, the HBsAg titers quantified by Alinity will be high in comparison with the titers quantified by Lumipulse, HISCL, and Elecsys quant II. There are many possible causes for the discrepancy in HBsAg titers, but the genotypic difference was considered to be a contributing factor. For example, the slope of the linear regression of CL AIA against Alinity was 1.025, indicating a good correlation of HBsAg titers. However, the slope of GTB was 0.5776, which is far off from 1.0, while the slopes of GTA, GTC, and GTD were close to or not too far from 1.0, indicating a marked difference by genotype. Other HBsAg kits also showed varying correlations between kits, depending on genotypes. Although HBsAg quantification kits evaluated in this study use polyclonal antibodies or a cocktail of monoclonal antibodies to capture and detect various HBsAg, the genotype-dependent differences in HBsAg quantification remain unresolved. Furthermore, HBsAg has a very wide range of measurement, and the titers of specimens in the RRP used in this study were distributed from lower than 1 IU/mL to the order of 10^5^, making it difficult to match quantified titers. The standardization of HBsAg assays is a challenge that remains to be addressed in future studies.

## Conclusions

We found that the *in vitro* diagnostic kits for HBV DNA have enough sensitivity and specificity for the diagnosis of HBV infection. HBV DNA titers are compatible among HBV DNA kits and are robust to HBV genotypes. HBsAg kits have enough specificity for the screening of HBV infection. The sensitivities of HBsAg kits varied, but 1 LFA kit has a high sensitivity which is equivalent to that of automated HBsAg kits. Unfortunately, HBsAg titers were not consistent among the kits, suggesting limitations for usage in the monitoring of HBV infection status. The issue of how to standardize HBsAg kits for quantification remains to be addressed.

## Electronic supplementary material

Below is the link to the electronic supplementary material.

Supplementary Material 1

## Data Availability

The datasets analyzed during the current study are not publicly available because the raw data measured by the manufacturers cannot be provided, but are available from the corresponding author on reasonable request.

## Abbreviations

CV: coefficient of variation
GT: genotype
HBsAg: Hepatitis B surface antigen
HBV: Hepatitis B virus
IS: International Standard
IU: International Units
LFA: lateral flow assay
RRP: regional reference panel
WHO: World Health Organization.
