# Supplementary material for: Performance evaluation of in vitro diagnostic kits for hepatitis B virus infection using the regional reference panel of Japan
Source: Virol J. 2023 May 10;20:93. doi: 10.1186/s12985-023-02054-7 (PMC10170722; doi:10.1186/s12985-023-02054-7)
Supplement: Supplementary file 1 — Supplementary Material 1 [file 12985_2023_2054_MOESM1_ESM.pdf]

Additional File

Performance Evaluation of In Vitro Diagnostic Kits for Hepatitis B Virus Infection Using the Regional Reference Panel of Japan

Haruka Momose, Asako Murayama, Norie Yamada, Keiji Matsubayashi,

Sahoko Matsuoka, Emi Ikebe, Madoka Kuramitsu, Masamichi Muramatsu,

Takanobu Kato, Isao Hamaguchi.

Table S1. HBV DNA kits evaluated in this study.

Kits	Manufacture	Method	Target	Dynamic range (log IU/mL)
ART HBV	Abbott Japan	qPCR	HBs	1.00–9.00
Alinity HBV				
CAP/CTM HBV v2.0	Roche Diagnostics	qPCR	preC/C	1.30–8.23
Cobas HBV				1.00–9.00
Aptima HBV	Hologic	TMA	HBs	1.00–9.00

qPCR; quantitative PCR, TMA; Transcription Mediated Amplification, HBs; hepatitis B surface, preC/C; hepatitis B precore/core region.

Table S2. HBsAg kits evaluated in this study.

HBsAg kits		Manufacture	Method	Cut off (IU/mL)
Quantitative	Lumipulse	FUJIREBIO	CLEIA	0.005
	Presto			
	HISCL	Sysmex	CLEIA	0.03
	CL AIA	Tosoh	CLEIA	0.03
	Accuraseed	FUJIFILM Wako	CLEIA	0.05
	Architect	Abbott Japan	CLIA	0.05
	Alinity			
	Elecsys quant II	Roche Diagnostics	ECLIA	0.05
Qualitative	ST AIA	Tosoh	FEIA	0.05
	STACIA	LSI Medience	CLEIA	N/A
	Centaur II	Siemens	CLIA	N/A
	Elecsys II	Roche Diagnostics	ECLIA	N/A
LFA	Determine	Abbott Diagnostics Medical	LFA	N/A
	Determine2			

CLIA; Chemiluminescent immunoassay, CLEIA; Chemiluminescent enzyme immunoassay, ECLIA; Electrochemiluminescence immunoassay, FEIA; Fluorescence enzyme immunoassay, LFA; Lateral flow assay, N/A; not applicable.

Figure S1.

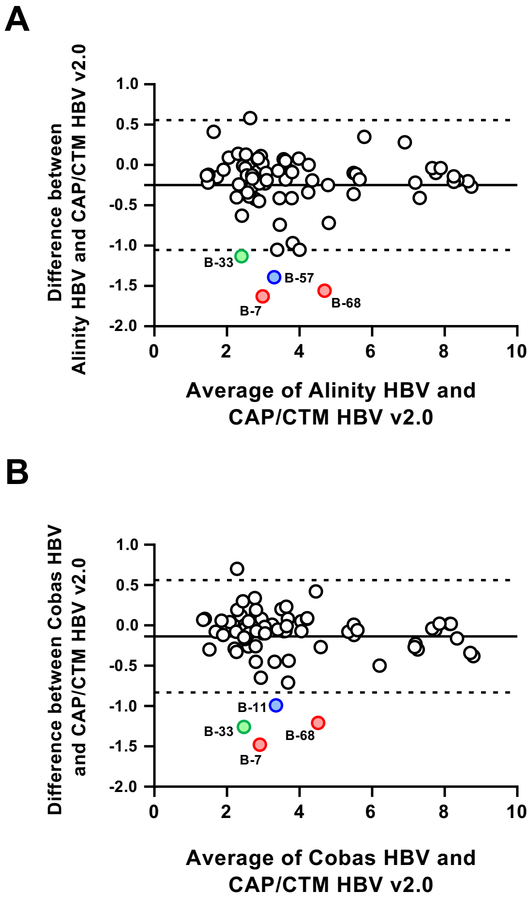

Figure S1. Deviation of HBV DNA titers quantified by CAP/CTM HBV v2.0.

Deviations of HBV DNA titers by CAP/CTM HBV v2.0 from Alinity HBV (A) and Cobas HBV (B) were analyzed by the Bland–Altman method. Specimens with significantly low titers in CAP/CTM HBV v2.0 are indicated by colored circles. Red; GTA, blue; GTB, green; GTC.

Figure S2.

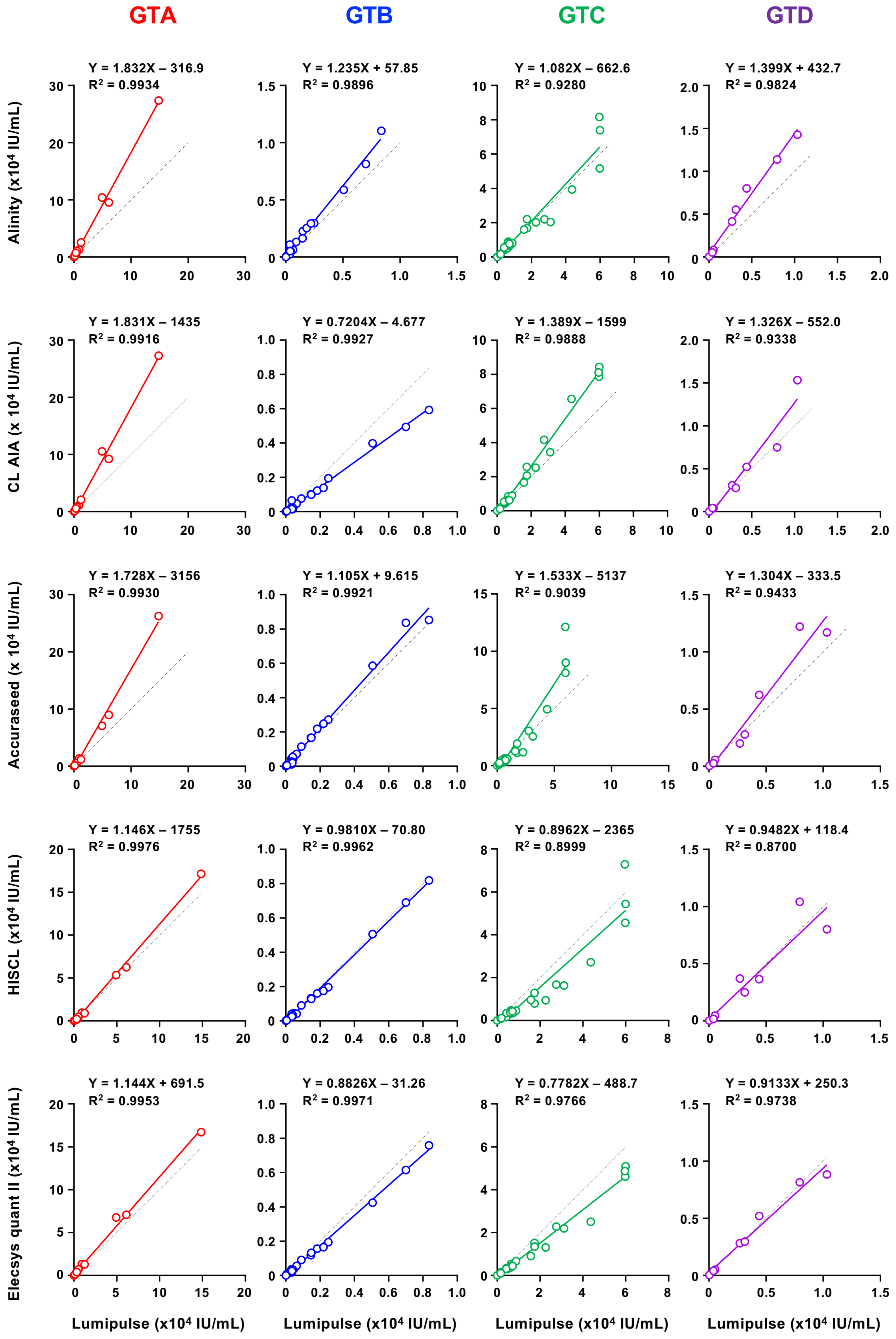

Figure S2. Genotype-dependent differences in HBsAg titers compared to Lumipulse.

Correlations of HBsAg titers of Alinity, CL AIA, Accuraseed, HISCL, and Elecsys quant II were analyzed against Lumipulse. Data and linear regression lines are shown in dots and lines, respectively. Dashed lines indicate a slope of 1.0.

Figure S3.

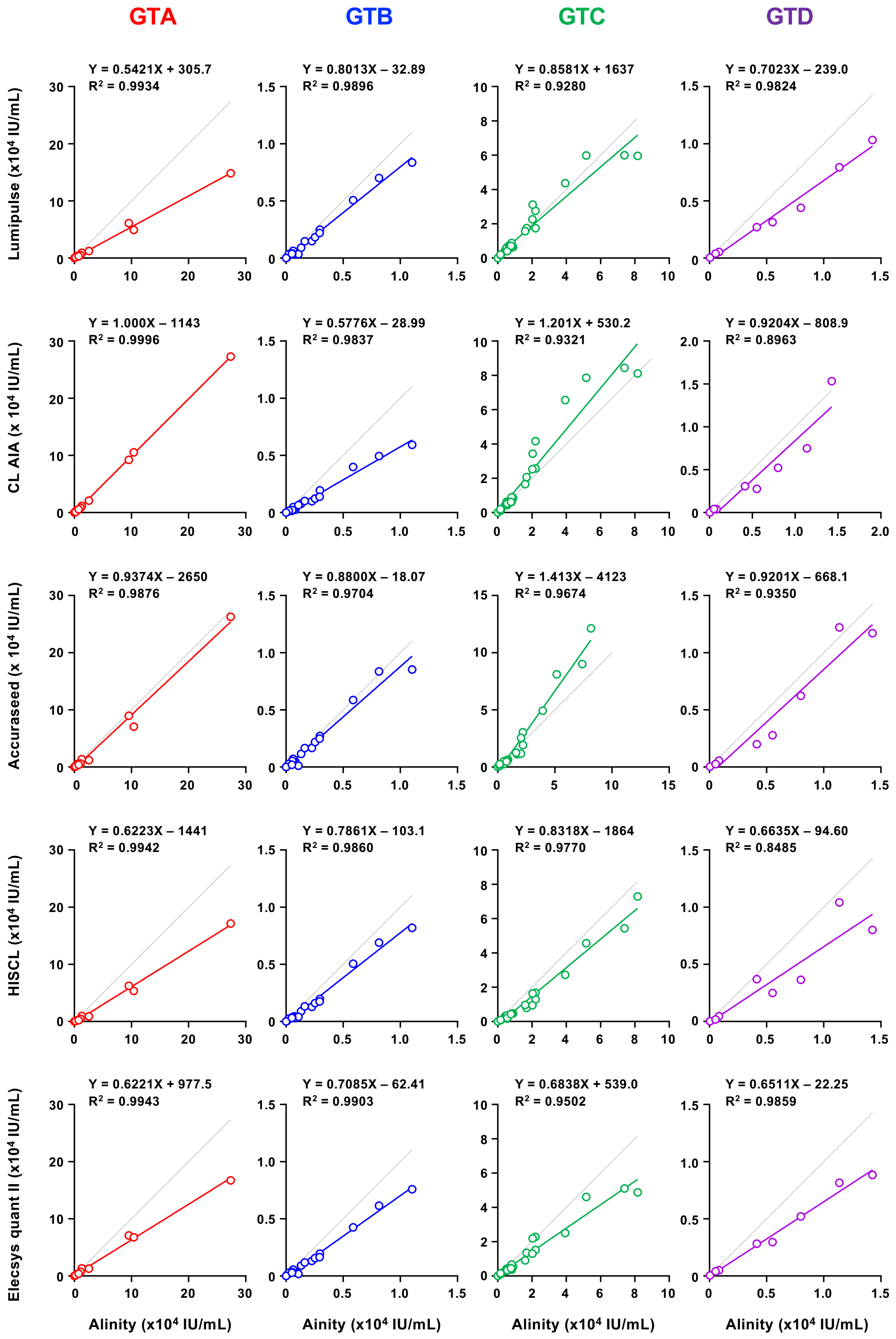

Figure S3. Genotype-dependent differences in HBsAg titers compared to Alinity.

Correlations of HBsAg titers of Lumipulse, CL AIA, Accuraseed, HISCL, and Elecsys quant II were analyzed against Alinity. Data and linear regression lines are shown in dots and lines, respectively. Dashed lines indicate a slope of 1.0.

Figure S4.

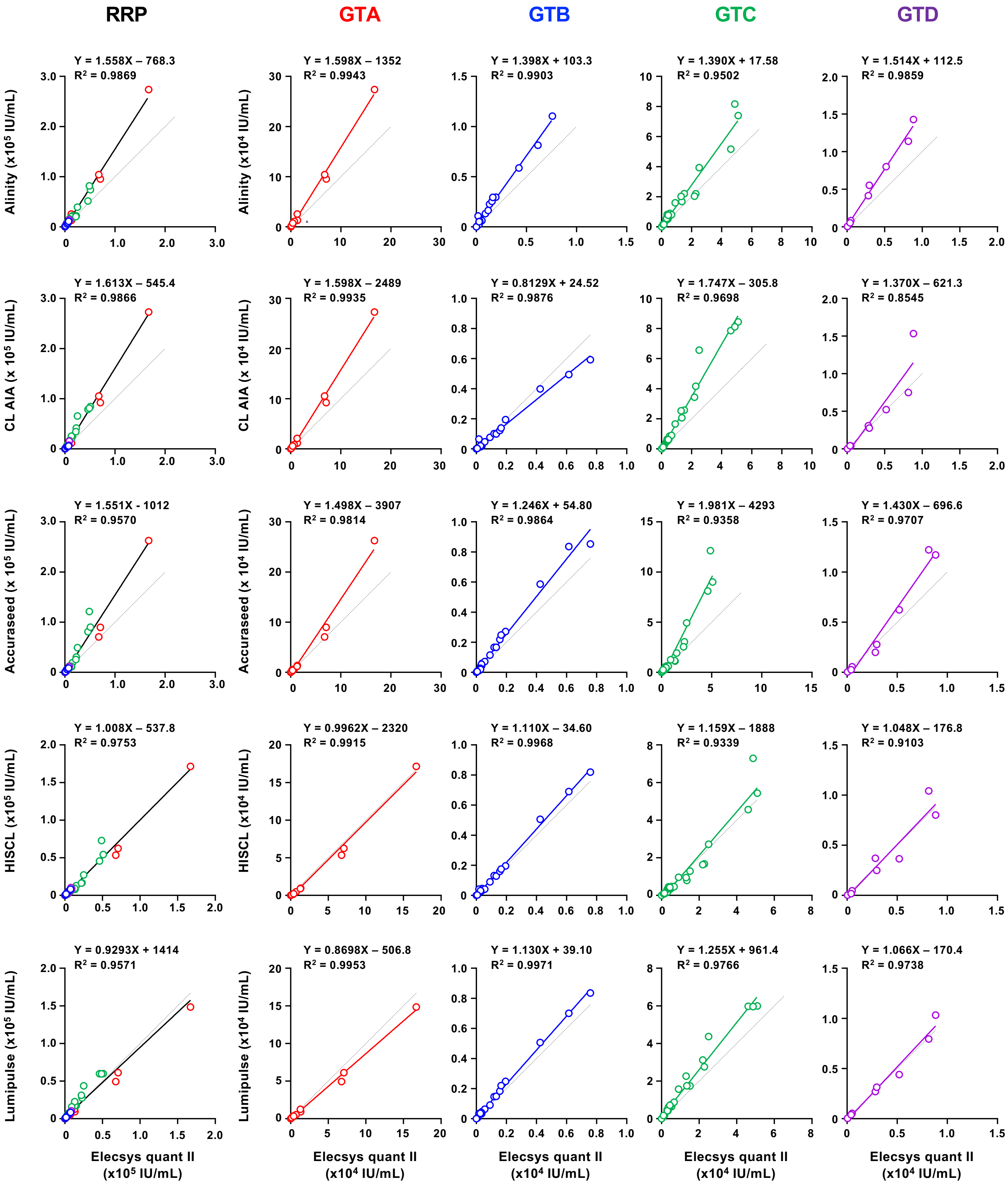

Figure S4. Genotype-dependent differences in HBsAg titers compared to Elecsys quant II.

Correlations of HBsAg titers of Alinity, CL AIA, Accuraseed, HISCL, and Lumipulse were analyzed against Elecsys quant II. Data and linear regression lines are shown in dots and lines, respectively. Dashed lines indicate a slope of 1.0.
